# Prevalence of the F508del variant in patients with cystic fibrosis in northeastern Brazil: clinical and laboratory factors

**DOI:** 10.36416/1806-3756/e20240145

**Published:** 2026-11-09

**Authors:** Evalto Monte de Araújo, Claudia Castro e Silva, Hildenia Balthazar Ribeiro Nogueira, Livia Cordeiro Bastos de Araújo, Lilia Cordeiro Bastos Silveira, Olivia Andrea Alencar Costa Bessa

**Affiliations:** 1. Serviço de Pneumologia Pediátrica, Hospital Infantil Albert Sabin - HIAS, Fortaleza (CE) Brasil.; 2. Serviço de Pneumologia Pediátrica, Hospital Infantil Albert Sabin - HIAS, Fortaleza (CE) Brasil.; 3. Serviço de Gastroenterologia Pediátrica, Hospital Infantil Albert Sabin - HIAS, Fortaleza (CE) Brasil.; 4. Serviço de Neonatologia, Maternidade-Escola Assis Chateaubriand - MEAC, Fortaleza (CE) Brasil.; 5. Curso de Medicina, Universidade Christus - UNICHRISTUS, Fortaleza (CE) Brasil.; 6. Faculdade de Medicina, Universidade de Fortaleza - UNIFOR, Fortaleza (CE) Brasil.

**Keywords:** Cystic fibrosis, Genotype, Phenotype, Prevalence

## Abstract

**Objective:**

To describe the genetic profile of patients with cystic fibrosis in northeastern Brazil and correlate it with clinical and laboratory characteristics.

**Methods:**

This was a cross-sectional study, with descriptive and analytical elements, of patients followed during 2021 at a referral center for cystic fibrosis in the Brazilian state of Ceará. We analyzed pathogenic variants of genes that play a role in cystic fibrosis and their correlations with clinical variables (gender, age, weight, height, BMI, and nutritional status), laboratory variables (microbiology and sweat test results), and medications in use.

**Results:**

Of a total of 86 patients evaluated, 85 underwent genetic testing. Homozygous F508del mutations were identified in 60%, and 95% had a single F508del allele. Class IV or V mutations were seen in 4.6%. The majority of the mutations were in functional class I, II, or III. We found that a homozygous F508del mutation was significantly associated with BMI (p < 0.019), nutritional status (p < 0.03), and a positive sweat test result (p < 0.049), as well as identifying a significant association between the microbiology result and patient age (p < 0.001).

**Conclusions:**

In our sample, the proportion of patients who were homozygous for the F508del mutation was well above the national and international averages, which makes those patients potentially eligible for treatment with at least one CF transmembrane conductance regulator modulator.

## INTRODUCTION

Cystic fibrosis (CF), initially described in 1938 by Dr. Dorothy Andersen as a significant cause of malnutrition and early death in children, is the most common autosomal recessive genetic disease among White individuals and presents as a multisystemic pathology.^1^ ^-^ ^3^ It results from a genetic alteration in the CF transmembrane conductance regulator (*CFTR*) gene, which encodes a protein also known as CFTR. This genetic alteration leads to changes in chloride and water transport across the cell membranes in various organs.^4^ ^,^ ^5^

Among the more than 2,000 genetic variants already described, the four most often studied in Brazil are F508del, G542X, G551D, and N1303K. Understanding the effect of the genetic variant on protein function is an essential step in the treatment of CF. For better characterization, a classification system was created, with six classes according to the amount/quality of CFTR protein function. The most common genetic variant worldwide is F508del, which is categorized as a class II mutation, meaning that there is a defect in the synthesis of the CFTR protein.^5^ ^-^ ^8^

Worldwide, the estimated incidence of CF is 1 in every 2,800-3,500 live births. In Brazil, that incidence is approximately 1 in every 10,000 live births.^6^

Mutations in the *CFTR* gene give CF a multisystemic nature, characterized by respiratory, gastrointestinal, and sweat gland manifestations. In the respiratory component, recurrent infections (sinusitis and pneumonia) predominate, which can result in irreversible lower airway changes collectively known as bronchiectasis. In the gastrointestinal context, there are varying degrees of exocrine pancreatic insufficiency, leading to a syndrome of malabsorption of macronutrients, micronutrients, and fat-soluble vitamins (A, D, E, and K), resulting in energy-protein malnutrition.^6^ ^-^ ^11^

A diagnosis of CF is initially suspected on the basis of the immunoreactive trypsinogen (IRT) level, determined during neonatal screening. In Brazil, CF screening was introduced into the National Neonatal Screening Program in 2001 by testing IRT levels in blood samples collected from newborns. The diagnosis is confirmed by a positive result on a sweat test (the gold standard), the identification of a relevant genetic variant, and the presence of at least one phenotypic characteristic of the disease.^8^ ^-^ ^12^ A correlation between genotype and phenotype is already evident in the literature, because it can be observed in patients with mutations that result in reduced or absent CFTR protein function who present early exocrine pancreatic insufficiency and lung disease that is more advanced.^13^

Despite recent, ongoing advances in diagnosis and therapy, CF continues to be associated with significant morbidity and mortality. Survival is related to early diagnosis and multidisciplinary follow-up at CF referral centers, as well as to the availability of advanced therapies for treating the underlying cause and emerging complications.^11^ ^-^ ^13^

The primary objective of this study was to describe the genetic profile of patients with CF treated at a tertiary referral center in northeastern Brazil. A secondary objective was to look for correlations among clinical and laboratory characteristics.

## METHODS

This was an observational (retrospective and prospective) cohort study, with descriptive and analytical elements, conducted from January to December of 2021 at the multidisciplinary Cystic Fibrosis Outpatient Clinic of the Hospital Infantil Albert Sabin (HIAS, Albert Sabin Children’s Hospital), a referral center for children and adolescents with rare and highly complex diseases, located in the city of Fortaleza, in the northeastern Brazilian state of Ceará.

We included children and adolescents who were diagnosed with CF on the basis of increased chloride ion concentrations on a sweat test. The tests were performed in the HIAS laboratory with the coulometry technique in a chloridometer (ChloroChek; ELITechGroup Inc, Logan, UT, USA), which quantitatively analyzes chloride levels in sweat. The diagnosis of CF is confirmed if the chloride level is above 60 mmol/L in two sweat tests or at least one genetic variant that is pathogenic for CF is identified.

The HIAS Cystic Fibrosis Outpatient Clinic is the referral center for CF in the state of Ceará and is the only public institution in the state that performs the sweat test. All pediatric patients with a positive sweat test are included in the HIAS follow-up protocol.

During the study period, there were over 100 patients registered at the HIAS Cystic Fibrosis Outpatient Clinic. The inclusion criteria were as follows: being ≤ 17 years of age; having undergone a sweat test; carrying at least one pathogenic genetic variant; presenting with clinical signs and symptoms consistent with CF; and having had at least three appointments during the study period. 

Data were collected from the multidisciplinary outpatient clinic records, patient medical records, and the Brazilian Cystic Fibrosis Registry. We employed a written questionnaire designed to collect information on clinical characteristics (signs, symptoms, and medications in use). We also collected the following data: anthropometric parameters (weight, height, and BMI); the results of laboratory tests (IRT, sweat test, lung function, and microbiology); the results of genetic testing (genetic variant); treatment regimen (antibiotic use); team structure (multidisciplinary or other); and sociodemographic characteristics (age and sex).

Genetic testing was conducted during regularly scheduled visits at the HIAS Cystic Fibrosis Outpatient Clinic. After a 30-min patient fast and oral hygiene, saliva samples were collected with a buccal swab. Mucosal secretions were collected from both sides of the mouth, after which the material was placed in a tube, stored, and transported at room temperature to a commercial laboratory (Mendelics, São Paulo, Brazil) for genetic panel analysis. Variant analysis was performed by mapping the genetic sequence to the Hg19 reference human genome with the BWA-MEM alignment tool and the Genome Analysis Toolkit (The Broad Institute of Harvard and MIT, Cambridge, MA, USA).

The World Health Organization (2006-2007) growth curves and the anthropometric data obtained from the children and adolescents in our sample were evaluated. We calculated Z scores for height/age, weight/age, weight/height, and BMI/age.

For the descriptive analysis, continuous variables are expressed as means, medians, standard deviations, and interquartile ranges, whereas discrete variables are expressed as absolute and relative frequencies. The chi-square test was used for comparing categorical variables. A probability level of p < 0.05 was adopted for all inferential procedures.

The study was approved by the Research Ethics Committee of the HIAS (Reference no. 4,547,442). All participating patients gave written informed consent.

## RESULTS

Of the 100 eligible patients, 86 were evaluated at the multidisciplinary HIAS Cystic Fibrosis Outpatient Clinic. Of those 86 patients, 43 (50%) were male. The mean age was 21.4 ± 31.5 months at the time of diagnosis and 9.46 ± 5.05 years at inclusion in the study.

All of the patients evaluated underwent a sweat test. The median sweat chloride level was 92 mmol/L (IQR, 31-140 mmol/L). Genetic testing was conducted for 85 patients, and the most prevalent variant was homozygous F508del (F508del/F508del), which was seen in 52 (61.2%) of those 85 patients. The F508del allele alone was present in 95% of the samples (Table 1). In addition, we identified some rare, less commonly described genetic variants, such as W1098C and 3849+10 kb C->T. One patient did not have a pathogenic genetic variant for CF, despite having a sweat chloride level > 60 mmol/L (Table 1).

**Table 1:** Genetic variants and sweat test results in patients with cystic fibrosis (N = 86).

Genetic variant	n (%)	CFTR class	Sweat chloride
			(mmol/L)*
F508del/F508del	52 (60.4)	II/II	94.7 ± 16.5
F508del/[unknown]	10 (11.6)	II/[unknown]	82.6 ± 23.4
F508del/3120+1G->A	3 (3.5)	II/I	89.0 ± 10.5
F508del/W1282X	3 (3.5)	II/I	96.3 ± 9.0
F508del/R1066C	2 (2.3)	II/II	91.0 ± 9.8
F508del/2184insA	2 (2.3)	II/I	99.0 ± 16.9
F508del/G542X	1 (1.2)	II/I	101.0
F508del/I1234V	1 (1.2)	II/IV	103.0
F508del/P205S	1 (1.2)	II/IV	80.0
F508del/S466X	1 (1.2)	II/I	111.0
F508del/S549R	1 (1.2)	II/III	108.0
F508del/W1098C	1 (1.2)	II/missense mutation^†^	104.0
F508del/Y1092X	1 (1.2)	II/I	96.0
F508del/3849+10 kb C->T	1 (1.2)	II/splice mutation^†^	52.0
F508del/3272-26A->G	1 (1.2)	II/V	99.0
P205S/G542X	1 (1.2)	IV/I	63.0
W1282X/L206W	1 (1.2)	I/II	69.0
5T/[unknown]	1 (1.2)	I/[unknown]	62.0
None	1 (1.2)	-	86.0
Not tested	1 (1.2)	-	XX.X

CFTR: cystic fibrosis transmembrane conductance regulator. *Where appropriate, sweat chloride data are presented as mean ± SD. ^†^Rare genetic variants, less described in the literature.

On average, the patients with the homozygous F508del mutation had lower BMIs and higher nutritional deficits than did those who were heterozygous for F508del. There was no significant difference between the two groups in terms of sex or age (Table 2).

**Table 2:** Sociodemographic and anthropometric characteristics of patients with cystic fibrosis, by F508del group.

Characteristic	Total	F508del group		p
		Homozygous	Heterozygous	
	(N = 86)	(n = 51)	(n = 35)	
Sex, n (%)				
Female	43 (50.0)	27 (52.9)	16 (45.7)	0.42
Male	43 (50.0)	24 (47.1)	19 (54.3)	
Age (years)				
Mean ± SD	9.46 ± 5.05	8.84 ± 4.98	10.45 ± 5.08	0.164
Median (IQR)	9 (6-13)	8.5 (4-13)	11 (6-14)	
BMI (kg/m2)				
Mean ± SD	16.68 ± 2.71	15.93 ± 2.22	17.74 ± 3.02	0.019
Median (IQR)	16.4 (14.8-18.5)	15.9 (13.9-16.9)	16.85 (15.7-18.6)	
Nutritional status, n (%)				
No data	21 (24.4)	13 (25.5)	8 (22.8)	0.03
No deficit	43 (50.0)	21 (41.2)	22 (62.8)	
Deficit	18 (20.9)	15 (29.4)	3 (8.6)	
Obesity	4 (4.6)	2 (3.9)	2 (5.7)	

The sweat chloride levels were significantly higher in the homozygous F508del group than in the heterozygous group. The proportion of patients with respiratory, gastrointestinal, or hepatic symptoms was also higher in the homozygous F508del group, although the difference was not statistically significant (Table 3). Three patients did not carry the F508del allele and were therefore not included in either group. Meconium ileus was observed in 7 (8.1%) of the 86 patients, and 5 of those patients were homozygous for F508del.

**Table 3:** Clinical and laboratory variables in patients with cystic fibrosis, by F508del group.

Variable	Total with data available	F508del group		p
		Homozygous	Heterozygous	
	(n)	(n = 52)	(n = 29)	
Clinical manifestations, n (%)				
Respiratory	80	49 (94.2)	28 (96.6)	0.21
Gastrointestinal	77	49 (94.2)	24 (82.8)	
Hepatic	11	7 (13.5)	4 (13.8)	
Inhaled antibiotics, n (%)				
Tobramycin	18	10 (19.2)	8 (27.6)	0.12
Colistin B	10	6 (11.5)	4 (13.8)	
Aztreonam	4	2 (3.8)	2 (6.9)	
Microbiology result, n (%)				
Negative	28	17 (32.7)	11 (37.9)	0.878
Intermittent PA	13	8 (15.4)	5 (17.2)	
Mucoid PA	30	18 (34.6)	12 (41.4)	
*Burkholderia cepacia*	5	3 (5.8)	2 (6.9)	
*Serratia* sp.	1	0 (0)	1 (3.4)	
SA	1	1 (1.9)	0 (0)	
Sweat chloride (mmol/L)				
Mean ± SD	91.71 ± 17.93	94.82 ± 16.68	87.03 ± 18.96	0.049
Median (IQR)	92 (80-104)	93 (83-106)	88.5 (76-101)	

PA: *Pseudomonas aeruginosa*; and SA: *Staphylococcus aureus*.

Gastrointestinal manifestations were present in nearly 90% of the patients. Sudam III staining for fecal fat and testing for fecal elastase were used in order to identify pancreatic insufficiency and steatorrhea. There was no statistical difference between the homozygous and heterozygous F508del groups.

Liver disease associated with CF was found in 14% of the patients. The main findings included elevated transaminases, an elevated international normalized ratio, and changes seen on ultrasound, such as a heterogeneous parenchyma. One patient had CF-related diabetes.

At our facility, pulmonary function testing is not performed in children under 6 years of age. The majority of the patients in our sample were over 6 years of age, and spirometry could not be performed during the study period due to the COVID-19 pandemic.

We found a positive correlation between age and the microbiology results (Table 4). As age increased, there was a significant increase in the prevalence of colonization by *Pseudomonas aeruginosa* or *Burkholderia cepacia*.

**Table 4:** Microbiology results in patients with cystic fibrosis, by age group.

Microbiology result	Age group			p
	< 6 years	6-10 years	> 10 years	
	n (%)	n (%)	n (%)	
Negative	11 (61.1)	10 (43.5)	5 (14.7)	
Intermittent PA	6 (33.3)	4 (17.4)	2 (5.8)	< 0.001
Mucoid PA	1 (5.6)	8 (34.8)	21 (61.7)	
*Burkholderia cepacia*	0 (0)	0 (0)	5 (14.7)	
*Serratia marcescens*	0 (0)	0 (0)	1 (2.9)	
SA	0 (0)	1 (4.3)	0 (0)	

PA: *Pseudomonas aeruginosa*; and SA: *Staphylococcus aureus*.

## DISCUSSION

Associations between genotypes and phenotypes in CF facilitate understanding of the broad spectrum of disease manifestations, which can vary from single-organ to multisystemic involvement and from mild to severe. Genetic variant analysis is essential for diagnosing and determining the prognosis of CF, especially in cases in which the sweat test results do not confirm the diagnosis. In our study sample, there were 2 patients with a sweat chloride level < 40 mmol/L and pathogenic genetic variants in both alleles, highlighting the importance of genetic testing even in cases with atypical sweat test results.^13^ ^,^ ^14^

Our finding that there was no statistically significant difference among the patients with CF in terms of sex distribution is in contrast with the most recent data from the Brazilian Cystic Fibrosis Registry, in which there was a slight predominance of males. The prevalence of the homozygous F508del mutation in our study sample is in keeping with data in the literature showing that this mutation is the most prevalent worldwide, having been shown to be present in approximately 66% of all CF patients.^15^

Of the 85 patients who underwent genetic testing in our study, 2 (2.4%) were found to carry the G542X mutation, together with the F508del mutation (G542X/F508del) in one and the P205S mutation (G542X/P205S) in the other. The proportion of CF patients with the G542X mutation in our sample (2.4%) is similar to the 2.3% reported for patients in the state of Bahia and lower than the 8.0% reported for patients in the state of São Paulo.^2^ ^,^ ^3^ The differences in prevalence among regions reflect the diverse population of Brazil and its history of colonization by peoples from Europe and elsewhere in the Americas.^2^ ^,^ ^3^ ^,^ ^9^ ^,^ ^13^

In our study sample, there was a high prevalence of genetic variants in classes I, II, or III. The mutations most commonly found were F508del, in 82 (96.5%) of the patients tested, W1282X, in 4 (4.7%), and 3120+1G->A, in 3 (3.5%). The three most prevalent mutations in our study sample (F508del, W1282X, and 3120+1G->A) are associated with disease phenotypes that are more severe. Other mutations, found with lower frequency, included R1066C, 2184insA, and P205S, in 2 patients (2.4%) each, and several others were found in only 1 (1.2%) each: I1234V, S466X, S549R, W1098C, Y1092X, 3849+10 kb C->T, 3272-26A->G, and L206W. The less common mutations identified in our patients are not well described in the literature.^2^ ^,^ ^3^ ^,^ ^7^ ^,^ ^9^ The P205S mutation is categorized as functional class IV and is associated with mild phenotypes and few gastrointestinal symptoms. The 3272-26A->G mutation belongs to functional class V and is also associated with mild disease phenotypes. The R1066C mutation is linked to severe disease phenotypes and therefore belongs to functional class II, similar to F508del.^(6 ,16)^

The most recent data from the Brazilian Cystic Fibrosis Registry show that over 30% of CF patients in Brazil were using inhaled tobramycin and almost 20% were using colistin,^17^ comparable to the 20% and 12%, respectively, in our study sample.^17^

Comparing the homozygous and heterozygous F508del groups, we found a positive association with BMI-for-age, nutritional status, and sweat chloride level. The majority of patients with CF develop pathogen colonization during their lives, the colonization being influenced by environmental factors and the mutation class. In the present study, a significant association was observed between patient age and the microbiology results, colonization with a mucoid strain of *P. aeruginosa* and colonization with *B. cepacia* both being more prevalent in patients over 10 years of age.^18^ ^,^ ^19^

Our study has some limitations. First, the numbers of outpatient visits were reduced during the COVID-19 pandemic. Nevertheless, we were able to demonstrate a high prevalence of the F508del mutation and its positive associations with certain clinical and laboratory factors. In addition, the pulmonary function laboratory at our facility was temporarily closed during the study period and lung function therefore could not be evaluated. However, we were able to evaluate the gastrointestinal and hepatic manifestations of CF. Furthermore, there could have been a selection bias, given that the study was conducted at a referral center for CF, and the severity of the disease was likely greater among the patients included in our sample, potentially leading to underdiagnosis of milder forms of the disease in the population. The ethnic diversity in Brazil, combined with the lack of comprehensive epidemiological data from all regions, highlights the diversity in genetic variants. Therefore, there is a need for studies conducted in other regions of the country and at other levels of care.

As new therapies targeting specific genetic mutation groups emerge, understanding the genetic profile of CF patients at referral centers can lead to more tailored approaches, improving patient outcomes and quality of life.
